# ASSOCIATION BETWEEN QUALITY OF LIFE AND DEPRESSION IN DYADS OF OLDER PRIMARY CARE PATIENTS AND FAMILY MEMBERS

**DOI:** 10.1093/geroni/igac059.2395

**Published:** 2022-12-20

**Authors:** Nicole Fowler, Anthony Perkins, Seho Park, Matthew Schroeder, Malaz Boustani

**Affiliations:** Indiana University School of Medicine, Indianapolis, Indiana, United States; Indiana University School of Medicine, Indianapolis, Indiana, United States; Indiana University School of Medicine, Indianapolis, Indiana, United States; Regenstrief Institute, Inc., Indianapolis, Indiana, United States; Indiana University School of Medicine, IU Center for Aging Research, Indianapolis, Indiana, United States

## Abstract

Familial dyads experience illness as an interdependent unit. We evaluate the association of quality of life (QOL), as measured by physical (PCS) and mental health component (MCS) scores, with depression in dyads of older primary care patients and a family member. This is a cross sectional, descriptive study where QOL and depression were measured concurrently in the dyad using baseline data from 1809 dyads enrolled in a trial testing the benefits and harms of Alzheimer’s disease and related dementias (ADRD) screening. QOL was measured with the SF-36, depression was measured with the PHQ-9, and the association of depression with QOL was examined using an actor-partner interdependence model with distinguishable dyads. Patient mean (SD) age was 73.7 (5.7) years; 53.1% women; 85.1% white; 13.4% black. Family member mean (SD) age was 64.2 (13) years; 67.7% women; 13.4% black. A patient’s spouse/partner were 64.8% of family members. After controlling for dyadic relationship and gender, significant actor effects of depression on PCS for patient (β= -1.39; p< 0.001) and family member (β =-0.954; p< 0.001), and significant partner effects of depression on PCS for patient (β=-0.15, p< 0.05) and family member (β =-0.18; p< 0.01). There were significant actor effects of depression on MCS for patient (β =-1.2; p< 0.001) and family member (β=-1.2; p< 0.001), but depression had a significant partner effect on MCS only for patient (β = -0.08; p< 0.05). Among dyads participating in an ADRD screening trial, dyads with higher depression had lower QOL. Family member depression was associated with decreased family member and patient QOL.

